# Multifunctional Metasurface Based on Cascaded Multilayer Modules

**DOI:** 10.3390/nano15201563

**Published:** 2025-10-14

**Authors:** Tongxing Huang, Shuai Huang, Zhijin Wen, Wei Jiang, Jianxun Wang, Yong Luo, Zeiwei Wu

**Affiliations:** School of Electronic Science and Engineering, University of Electronic Science and Technology of China, Chengdu 611731, China; tongxinghuang@std.uestc.edu.cn (T.H.);

**Keywords:** metasurface, multifunctional, multilayer modules, cascaded

## Abstract

This paper proposes a novel design method for multifunctional modular metasurfaces based on cascaded multilayer modules. Strong electromagnetic coupling between cascaded modules and balanced interface impedance achieved through optimized resonator configurations enable broadband operation. By pairwise cascading of the three modules to maximize utilization and achieve maximum channel count, the system realizes comprehensive electromagnetic wavefront manipulation across 4 broadband frequency ranges, demonstrating diverse functionalities including orbital angular momentum beam generation, polarization conversion, beam splitting, and radar cross-section reduction with 7 operational channels: two reciprocal co-polarized transmission channels at 14–20.7 GHz, individual reflection channels in +*z* and −*z* spaces at 32–38 GHz, two reciprocal cross-polarized transmission channels at 11.9–13.2 GHz, and a reflection channel in −*z* space at 20–28 GHz, spanning both transmission and reflection modes. The proposed cascading method is accomplished through direct attachment assembly, avoiding air coupling while enabling rapid installation and fast functional switching, providing flexibility for multifunctional electromagnetic wave control applications.

## 1. Introduction

Metasurfaces are two-dimensional planar materials composed of periodically arranged subwavelength artificial structural units have attracted widespread attention in the fields of optics and electromagnetics over the past decade [1,2,3]. With their precise control capabilities over electromagnetic wave amplitude [4,5], phase [6,7], and polarization [8,9], metasurfaces have demonstrated tremendous potential in numerous application areas, including beam shaping [10], holographic imaging [11], perfect absorption [12], and orbital angular momentum (OAM) vortex beam generation [13]. Particularly in cutting-edge technologies such as wireless communications, radar systems, optical imaging, and quantum information processing, metasurface devices are gradually transitioning from laboratory research to practical applications, becoming a key force for the development of next-generation optoelectronic technologies [14,15,16].

The growing complexity of modern electromagnetic applications has created an urgent need for versatile devices that can perform multiple functions simultaneously. Traditional single-function metasurfaces are increasingly insufficient to meet these evolving requirements. Multifunctional metasurfaces represent a key technological advancement in addressing this challenge, enabling the integration of various electromagnetic functionalities while enhancing both system efficiency and design flexibility [17,18,19,20]. Reconfigurable metasurfaces represent one of the most efficient approaches for rapidly implementing multifunctional capabilities, with dynamic control schemes based on active devices such as PIN diodes and varactor diodes becoming mainstream research directions [21,22,23,24,25]. However, the integration of numerous active devices significantly increases manufacturing costs and system complexity, while the insertion losses introduced by these devices reduce system efficiency and affect reliability in harsh environments. In contrast, modular cascading or stacking metasurfaces have emerged as another highly efficient and feasible method for achieving multifunctional, multichannel, and composite electromagnetic control [26]. Ref. [27] employed dense vertical stacking of independent metasurfaces with different materials for each layer, successfully achieving multispectral achromatic metalenses and eliminating chromatic aberrations in the visible range [28]. Utilized multilayer plasmonic nanoantennas to realize nonlinear holography, enabling background-free image formation at harmonic frequencies. Ref. [29] proposed a structure of N cascaded dual-channel metasurfaces to achieve 2^N switchable channels without intrinsic loss or cross-talk for certain functionalities. Ref. [30] presented the concept of modular radiating systems based on tightly cascaded transmit arrays, achieving OAM mode interconversion across multiple modes (l = 0, ±1, ±2) through cascaded module interactions. Ref. [31] utilized cascaded metasurfaces for generating switchable spatial light distributions through multiple pixel-scale alignment strategies between two metasurface pieces, realizing various diffraction patterns under different matching situations by combining Dammann optimization and gradient descent methods. However, existing metasurface designs are typically limited to narrow bandwidth ranges with restricted available frequency bands and single functionality. Most designs employ air-coupled cascading approaches, which are disadvantageous for applications in complex electromagnetic environments due to environmental instabilities and structural vulnerabilities.

**Figure 1:**
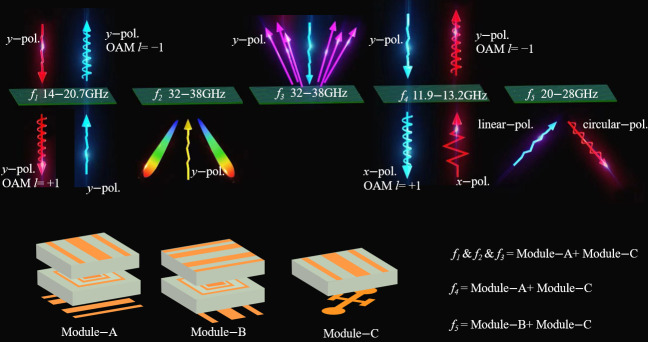
Schematic diagram of a modular cascaded multifunctional metasurface.

This study proposes a novel modular cascading metasurface approach that realizes multi-bands with broadband characteristics and diverse functional switching capabilities. The method features zero-spacing cascading through direct attachment assembly, which eliminates interlayer air coupling and ensures rapid, effective, and stable multifunctional switching operations. Three distinct types of multilayer modules (Module-A, -B, and -C) are designed and assembled through three different cascading configurations to achieve 5 electromagnetic wavefront manipulation functions across 4 independent broadband frequency ranges, demonstrating 7-channel control capability as shown in Figure 1. Specifically, the Module-A and Module-B cascade achieves co-polarized transmission OAM with two reciprocal channels at 14–20.7 GHz, co-polarized reflection dual-beam with individual channels in +*z* and −*z* spaces at 32–38 GHz, and RCS reduction. The Module-A and Module-C cascade enables cross-polarized transmission OAM with two reciprocal channels at 11.9–13.2 GHz, while the Module-B and Module-C cascade realizes linear-to-circular polarization conversion with a reflection channel in −*z* space at 20–28 GHz. This modular architecture provides flexibility and reliability for multi-band, multichannel electromagnetic wave manipulation in engineering applications.

## 2. Design and Operating Principle

Cascaded metasurfaces enable multifunctional electromagnetic operations through the integration of multiple functional layers. However, achieving independent control over multiple transmission and reflection channels presents formidable challenges due to intrinsic inter-channel coupling effects. The electromagnetic interactions between different channels inevitably introduce mutual interference, making it extremely difficult to establish multiple independent transmission-reflection pathways. Furthermore, interlayer electromagnetic coupling induces resonant frequency deviations from designed specifications and generates undesired phase perturbations [32,33]. These coupling-induced distortions significantly compromise the performance of cascaded structures, presenting critical obstacles for realizing independent multi-channel full-space electromagnetic manipulation.

The proposed approach addresses these fundamental challenges through a sophisticated modular cascading strategy that enables independent control of multiple transmission and reflection channels across full space. The secondary phase adjustment strategy effectively compensates for coupling-induced phase mismatches while simultaneously balancing impedance conditions to enable broadband characteristics. By implementing strategic modular cascading, the design successfully decouples the electromagnetic responses of different channels, achieving independent manipulation of multiple transmission-reflection pathways throughout the entire spatial domain. Furthermore, by configuring these three modules in pairwise combinations, the design maximizes channel utilization efficiency, enabling multichannel switching capabilities with minimal hardware redundancy. This modular architecture not only mitigates inter-channel coupling effects but also realizes independent control of multiple transmission–reflection channels in full space while optimizing the functional diversity-to-component ratio.

### 2.1. Multi-Channel Regulation in Full-Spacec

**Figure 2:**
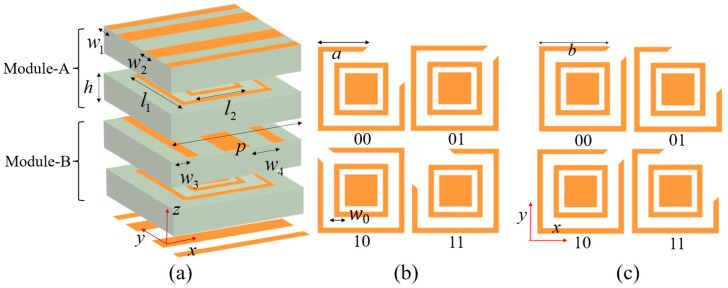
Schematic diagram of the cascaded modular metasurface unit cell structure. (**a**) 3D structure; (**b**) four coding states of Module-A; (**c**) four coding states of Module-B.

The structures of Module-A and Module-B are illustrated in Figure 2, which are multilayer unit-cell with orthogonal identical grating structures. The modules are intimately stacked with zero interlayer spacing, eliminating air gaps to suppress unwanted air coupling effects that can compromise the inter-cavity electromagnetic interaction. Both modules are constructed using F4B dielectric substrates with a relative permittivity of 2.65 and a thickness of *h* = 1 mm, arranged in a unit cell periodicity of *p* = 3.5 mm. The symmetric grating configurations ensure consistent polarization-selective responses. For a *y*-polarized wave incident along the −*z* direction, it is first converted to *x*-polarized wave by Module-A, then this *x*-polarized wave is converted back to *y*-polarized wave when passing through Module-B and transmitted through the bottom grating, ultimately achieving co-polarized transmission functionality. Due to structural symmetry, when a *y*-polarized wave is incident along the +*z* direction, it similarly undergoes dual polarization conversion and transmits a *y*-polarized wave through the top grating. The intermediate resonant layers of Module-A and Module-B are shown in Figure 2b,c, respectively, composed of split-ring resonators (SRRs), isolation rings, and central rectangular patches. The dimensions of the SRR can be controlled to modulate the transmission phase. The two-layer SRRs are designed for 2-bit phase quantization, where the unit cell supports digital phase encoding with four distinct states. The 2-bit phase encoding inherently requires four different phase states, which are realized through four distinct unit cell dimensional configurations in the designed metasurface. These different dimensional configurations are encoded using 2-bit binary representation (“00”, “01”, “10”, “11”), where each bit corresponds to a specific geometric parameter setting. The parameter a and parameter b values for these configurations are as follows: “00”: *a* = 2.1 mm, *b* = 2.9 mm; “01”: *a* = 2.9 mm, *b* = 2.1 mm; “10”: *a* = 2.1 mm, *b* = 2.9 mm; and “11”: *a* = 2.9 mm, *b* = 1.9 mm, respectively. The other key parameters are as follows: *l*_1_ = 3.2 mm, *l*_2_ = 2.2 mm, *w*_0_ = 0.1 mm, *w*_1_ = 0.2 mm, *w*_2_ = 0.5 mm, *w*_3_ = 0.35 mm, and *w*_4_ = 1.4 mm. The widths of the two grating layers are designed to be complementary, satisfying *w*_1_ + *w*_2_ + *w*_3_ + *w*_4_ = *p*.

**Figure 3:**
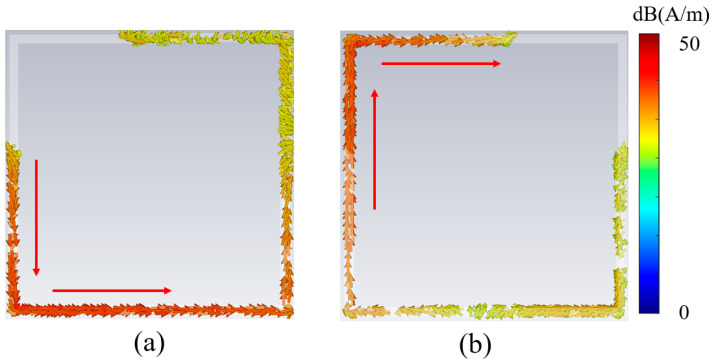
Simulation results of current distribution and direction in the cascaded Module-A and Module-B at 17 GHz. (**a**) Surface current distribution of SRR in Module-A; (**b**) surface current distribution of SRR in Module-B.

**Figure 4:**
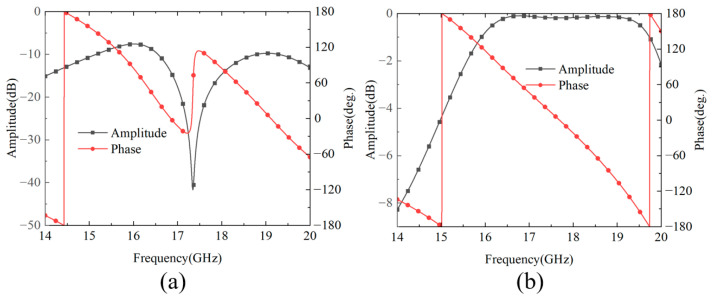
Simulated amplitude and phase responses. (**a**) single SRR module configuration; (**b**) dual SRR module configuration.

The phase modulation mechanism in the cascaded configuration operates via a two-stage current distribution process that enables electromagnetic phase control. Figure 3 shows the simulation results of the induced current distribution of the SRR structure at 17 GHz. In the first stage, incident electromagnetic waves interact with the SRR embedded within Module-A, inducing both transverse and longitudinal current components within the SRR structures, as demonstrated in Figure 3a. The effective current path length, which is governed by both the geometric parameter of arm length a and the orientation of the SRR, determines the magnitude of phase shift during this initial modulation stage. Subsequently, the intermediate grating structure performs polarization-selective transmission, allowing only the *x*-polarized field component to propagate toward Module-B. In the second modulation stage, this transmitted field undergoes further interaction with the SRR incorporated in Module-B, thereby generating additional transverse and longitudinal current distributions as illustrated in Figure 3b. The arm length parameter *b* within Module-B determines the effective current path length, thereby controlling the magnitude of secondary phase modulation and enabling cumulative phase control through the cascaded architecture. Furthermore, the SRR structures in both modules balance the impedance conditions at the inter-module coupling interface through electromagnetic coupling mechanisms. When only a single module incorporates SRR, as illustrated in Figure 4a, impedance discontinuity occurs at the coupling interface, resulting in a reflection-dominated regime for the cascaded modules at 17.5 GHz. When both modules contain SRR (Figure 4b), the symmetric SRR configuration establishes strong electromagnetic coupling between the dual resonator structures according to coupled resonator theory. This inter-module SRR coupling creates balanced impedance matching conditions and transforms the system from the reflection-dominated transmission regime. At zero separation distance, the coupling coefficient reaches its maximum value, thereby achieving maximum operational bandwidth.

**Figure 5:**
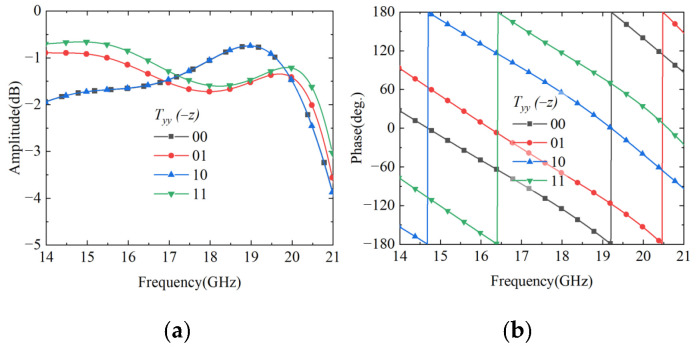
Transmission characteristics of the cascaded Module-A and Module-B under −*z* direction incidence. (**a**) Transmission amplitude; (**b**) transmission phase.

To validate the performance of the proposed cascade module unit, the performance is simulated using CST Microwave Studio. In the simulation setup, two Floquet ports are configured in the ±*z* directions, while unit cell boundary conditions are applied in the *x* and *y* directions to simulate a periodic array environment. The simulation results are presented in Figure 5. When a *y*-polarized wave is incident along the −*z* direction, the amplitude response of the co-polarized transmission coefficient Tyy maintains a high level within the operating frequency band, with a 3 dB transmission bandwidth of 14–20.7 GHz, corresponding to a relative bandwidth of 39.3%. From the phase response perspective, the transmission phases of the four coding states exhibit progressive characteristics. Although this is slightly increased compared to the single-module structure, it remains within an acceptable range and can meet practical application requirements. When a *y*-polarized wave is incident along the +*z* direction, the transmitted wave exits from the top grating, and its S-parameter characteristics are completely identical to those when the *y*-polarized wave is incident along the −*z* direction.

**Figure 6:**
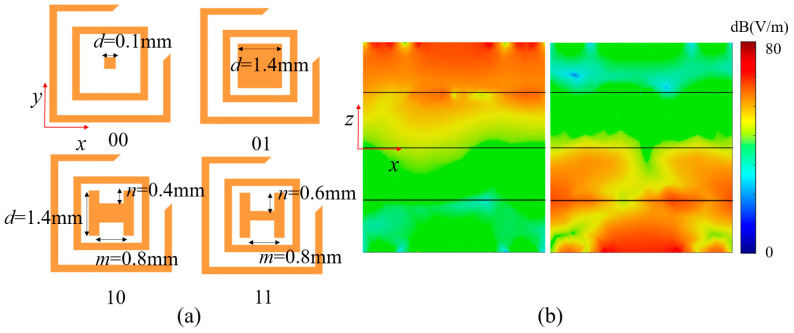
(**a**) 2-bit encoding configurations for Ka-band reflection phase control; (**b**) corresponding electric field intensity distributions at 35 GHz.

**Figure 7:**
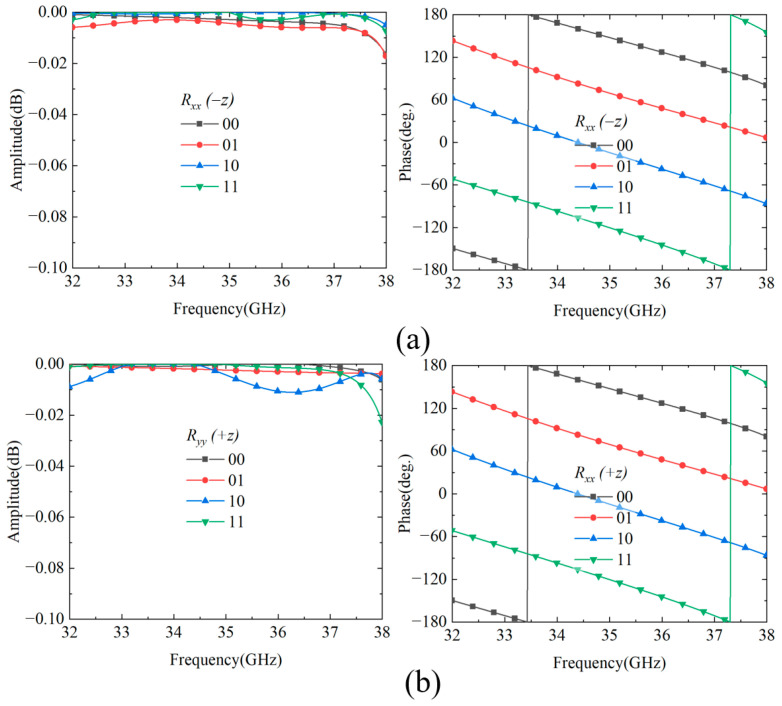
Reflection characteristics of cascaded Module-A and Module-B. (**a**) Reflection coefficients under −*z* direction incidence; (**b**) reflection coefficients under +*z* direction incidence.

To realize 2-bit co-polarized reflection phase modulation, the rectangular patch structure is enhanced to a combination of rectangular patch and H-shaped double-ridge structure by adjusting geometric dimension parameters, as shown in Figure 6a. The four coding states are distinguished through different size combinations of parameters *d*, *m*, and *n*. The specific parameter configurations are as follows: state “00” corresponds to *d* = 0.1 mm; state “01” corresponds to *d* = 1.4 mm; state “10” corresponds to *d* = 1.4 mm, *m* = 0.8 mm, *n* = 0.4 mm; state “11” corresponds to *d* = 0.6 mm, *m* = 0.8 mm, and *n* = 0.6 mm. Since the cascaded modular structure maintains polarization isolation characteristics, the upper and lower reflection resonators have identical dimensions for the four coding states, independently controlling the reflection phase responses in the +*z* and −*z* half-spaces, respectively. Figure 6b presents the simulation results demonstrating the independence of reflection control in the +*z* and −*z* half-spaces at 35 GHz. The electric field distribution contours clearly demonstrate that electromagnetic waves incident from the −*z* direction primarily excite the upper reflection resonator with intense field response, while the lower resonator remains largely inactive. Conversely, waves incident from the +*z* direction predominantly activate the lower reflection resonator with significant field concentration, while the upper resonator exhibits minimal response. This spatially selective electric field distribution validates that the cascaded modular structure, while maintaining polarization isolation characteristics, enables independent control of the reflection phase responses in the +*z* and −*z* half-spaces by the upper and lower reflection resonators, respectively, thereby ensuring the effectiveness of bidirectional independent modulation. For *y*-polarized waves, the cascaded structure demonstrates excellent bidirectional reflection performance. Under −*z* incidence, as shown in Figure 7a, all four coding states achieve reflection amplitudes exceeding −0.04 dB across 32–38 GHz (17.1% bandwidth) with ideal progressive phase distribution for 2-bit control. Similarly, as shown in Figure 7b, +*z* incidence maintains high reflection efficiency (>−0.02 dB) with consistent phase characteristics, confirming the system’s bidirectional stability and 2-bit phase control capability.

### 2.2. Bidirectional Cross-Polarization Transmission Regulation

**Figure 8:**
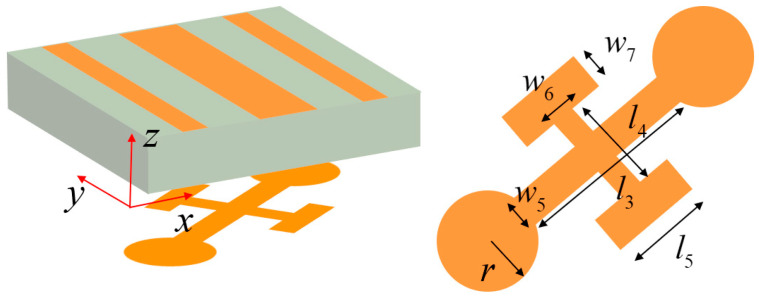
The structure of Module-C.

**Figure 9:**
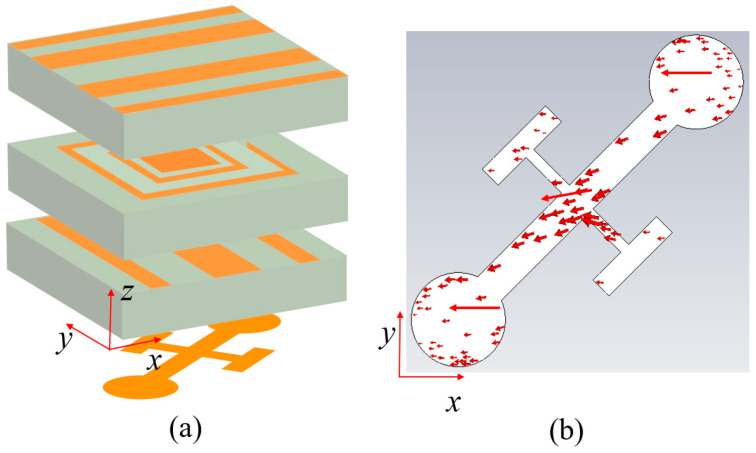
(**a**) Structural configuration of cascading Module-A and Module-C. (**b**) Simulated surface current distribution and direction of Module-C at 12.5 GHz.

**Figure 10:**
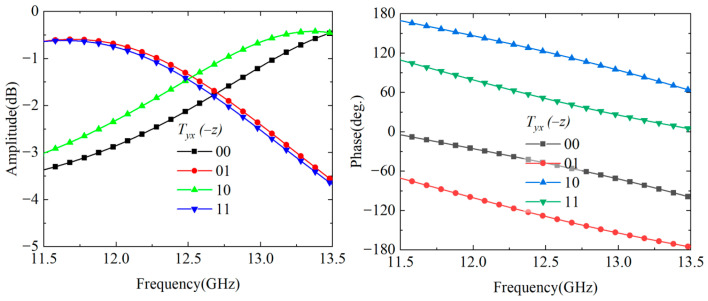
Simulated 2-bit cross-polarized transmission amplitude and phase for case of cascading Module-A and Module-C.

To achieve more diverse cascaded control effects with a single-layer grating structure. As shown in Figure 8, Module-C employs a design to maximize the utilization of Module-A. We designed Module-C with an asymmetric upper–lower layer design: the upper grating maintains consistency with the lower grating of Module-A, with the grating structure closely adhered to a 1 mm-thick F4B dielectric substrate; the lower metallic layer adopts a cruciform cross structure, where the long arm ends are connected to circular structures with radius *r*, and the short arms are connected to parallel rectangular patches. The key geometric parameters are set as follows: *l*_3_ = 2 mm, *l*_4_ = 1.2 mm, *l*_5_ = 0.8 mm, *w*_5_ = 0.3 mm, *w*_6_ = 0.1 mm, and *w*_7_ = 0.2 mm. Figure 9a presents the overall model after cascading Module-A and Module-C. When a y-polarized incident wave enters from the top grating along the −*z* direction, the electromagnetic wave first interacts with Module-A, achieving conversion from *y*-polarization to *x*-polarization and transmitting to Module-B. Subsequently, the tilted cruciform structure performs secondary modulation. As illustrated in Figure 9b, the induced current generated at 12.5 GHz is primarily concentrated in the long arms and two circular structures, with the current direction exhibiting transverse distribution. This frequency shift results from the cruciform resonator in Module-C establishing a novel induced current distribution when cascaded with Module-A, fundamentally reconfiguring the electromagnetic coupling mechanism and effective impedance characteristics of the system. As illustrated in Figure 9b, the induced current generated at 12.5 GHz is primarily concentrated in the long arms and two circular structures, with the current direction exhibiting transverse distribution, ultimately transmitting as an *x*-polarized wave to achieve cross-polarization transmission. When an *x*-polarized wave is incident along the +*z* direction, it can similarly be converted to a *y*-polarized wave and transmitted through the top grating, achieving bidirectional operating characteristics. Regarding phase control, the two-unit cascade primarily relies on the Module-A’s SRR to achieve 2-bit phase modulation functionality, while the cross cruciform structure undertakes phase correction. Specifically, for phase coding states “00” and “10”, the circular structure radius *r* is adjusted to 0.5 mm; for phase coding states “01” and “11”, the circular structure radius *r* is adjusted to 0.42 mm. Figure 10 presents the simulation results of 2-bit phase-controlled cross-polarization transmission when a *y*-polarized wave is incident along the −*z* direction. The simulation data reveals that the cascaded units achieve effective cross-polarization transmission within the frequency range of 11.9–13.2 GHz, with a 3 dB bandwidth of approximately 1.3 GHz and a relative bandwidth of 10.4%. The four phase states exhibit excellent progressive phase characteristics, with a maximum phase error of 24°, satisfying the requirements for 2-bit phase modulation. Additionally, the cross-polarization transmission S-parameters for *x*-polarized waves incident along the +*z* direction are identical to those for *y*-polarized waves incident along the −*z* direction.

### 2.3. Linear-Circular Polarization Conversion

**Figure 11:**
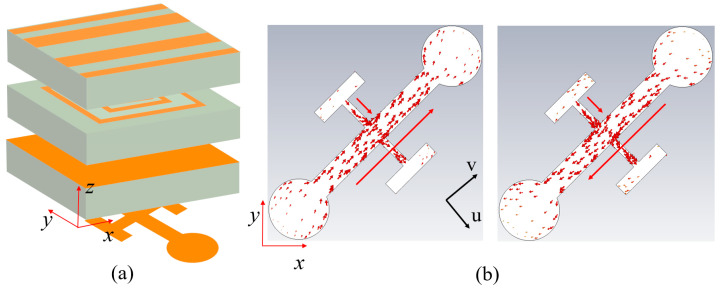
(**a**) Structural configuration of cascading Module-B and Module-C at 25 GHz. (**b**) Simulated surface current distribution and direction of a Module-C.

**Figure 12:**
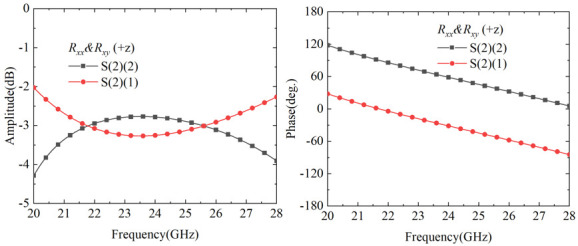
Simulated reflection amplitude and phase for case of cascading Module-B and Module-C.

As shown in Figure 11a, after rotating the additional unit by 90° and cascading it with Module-B, the complementary structural characteristics of the two grating layers enable the grating layer to function equivalently as a metallic ground plane. As illustrated in Figure 11b, at 25 GHz, when a linearly polarized wave is incident along the +*z* direction, the geometric asymmetry of the tilted cruciform structure excites orthogonal induced current components along the *u* and *v* directions: the long arm structure generates a dominant current in the +*v* direction, while the short arm structure excites bidirectional current distribution in the ±*u* directions. Based on vector synthesis principles, the superposition of +*v* direction current and +*u* direction current produces an *x*-polarized component, while the superposition of +*v* direction current and −*u* direction current produces a *y*-polarized component. When these two orthogonal components satisfy the conditions of equal amplitude and phase quadrature (90° phase difference), the cascaded structure achieves linear-to-circular polarization conversion functionality. The simulation validation shown in Figure 12 demonstrates that within the broadband range of 20–28 GHz (33.3% relative bandwidth), both the *y*-polarized reflection component S(2)(2) and *x*-polarized reflection component S(2)(1) maintain stable amplitudes around −3 dB, with the phase difference between the two polarization components precisely maintained at 90° and phase error controlled within 1°. This fully confirms that the cascaded structure can achieve broadband linear-to-circular polarization conversion applications. The proposed Module-C brings significant advantages to the cascaded metasurface system. By employing a different resonator design—specifically the cruciform cross structure—compared to the split-ring resonators in Module-A, Module-C creates a resonance frequency shift that establishes a new transmission passband in the 12–13 GHz range. Furthermore, the flexible utilization of the grating structure maximizes the resonator’s potential, enabling the cascaded system to achieve polarization conversion functionality in the 20–28 GHz range.

## 3. Metasurface Design and Results

### 3.1. Multifunctional Phase Encoding

Based on the designed three-module unit cells, a cascaded combination strategy enables multifunctional control of electromagnetic wavefronts, supporting multi-channel and multi-frequency dynamic switching. According to different cascaded configurations, the system can achieve 2 transmission functions and 3 reflection functions, specifically the following: *f*_1_ reciprocal co-polarization transmission (14–20.7 GHz), f_2_ reflection in −*z* space (32–38 GHz), *f*_3_ reflection in +*z* space (32–38 GHz), *f*_4_ reciprocal cross-polarization transmission (11.9–13.2 GHz), and *f*_5_ linear-to-circular polarization conversion in −*z* space (20–28 GHz).

**Figure 13:**
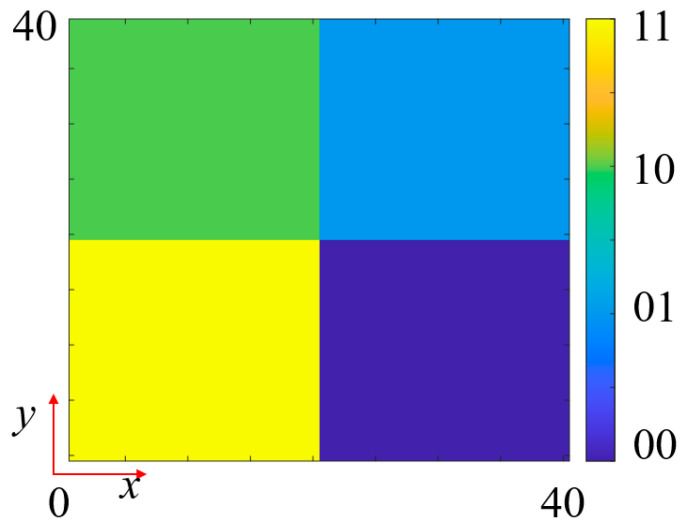
Phase encoding pattern for *f*_1_, *f*_4_ function with *l* = +1 OAM beam generation using 2-bit quantization.

The designed cascaded modules are arranged in a 40 × 40 array configuration to form the complete metasurface aperture. To achieve multifunctional control capabilities, different phase encoding schemes are employed for various channels. For *f*_1_, an OAM beam encoding approach is adopted with the vortex beam mode number set to 1. The required phase distribution of the OAM beam can be expressed as follows:

(1) $$\varphi_{OAM}=l\tan^{-1}\frac{y_{mn}}{x_{mn}},$$

where *l* is the OAM mode, and *x_mn_* and *y_mn_* are the positions of the (*m*, *n*)th unit cell. Figure 13 shows the calculated 2-bit quantized OAM beam (*l* = +1) phase coding pattern. Since *f*_4_ achieves phase modulation through SRR in Module-A, its phase encoding remains consistent with *f*_1_, thereby generating transmitted first-order OAM beams under different frequency bands and polarization conditions.

**Figure 14:**
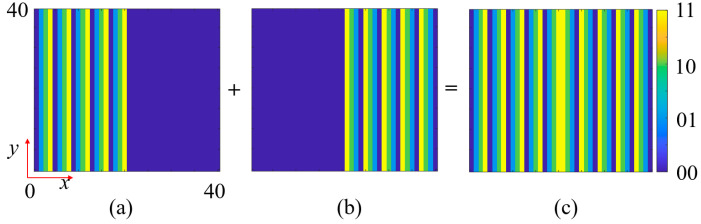
Reflected dual-beam coding of *f*_2_. (**a**) Gradient coding in the left half of the *x*-axis. (**b**) Gradient coding in the right half of the *x*-axis. (**c**) Superimposed metasurface coding pattern.

**Figure 15:**
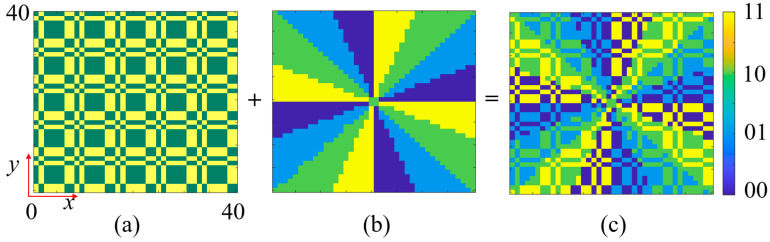
RCS reduction coding for *f*_3_. (**a**) 90° gradient diffusion coding pattern. (**b**) 4th-order OAM beam coding pattern. (**c**) Superimposed metasurface coding pattern.

For *f*_2_, a reflected dual-beam functionality is designed using a segmented encoding gradient phase scheme, as shown in Figure 14. Two 90° gradient phase encodings along the +*x*-axis and −*x*-axis are synthesized. The left and right halves of these two encoding patterns are extracted and combined to obtain the final phase encoding pattern. For *f*_3_, a novel RCS reduction electromagnetic wavefront control scheme is designed by superimposing a 90° gradient diffusion phase and a fourth-order OAM phase. Figure 15a presents the 90° gradient diffusion encoding pattern, Figure 15b shows the fourth-order OAM encoding pattern, and their superposition yields the final RCS reduction encoding (Figure 15c). This superposition strategy fully exploits the capability of gradient diffusion encoding to disperse reflected wave energy across multiple angular directions. Additionally, the fourth-order OAM beam, due to its helical phase structure, forms an annular intensity distribution in the far field with lower energy in the central region, further reducing backscattering intensity. The synergistic effect of these two mechanisms significantly reduces the radar cross-section in the detection direction, achieving high-performance monostatic RCS reduction.

### 3.2. Simulated and Test Results

**Figure 16:**
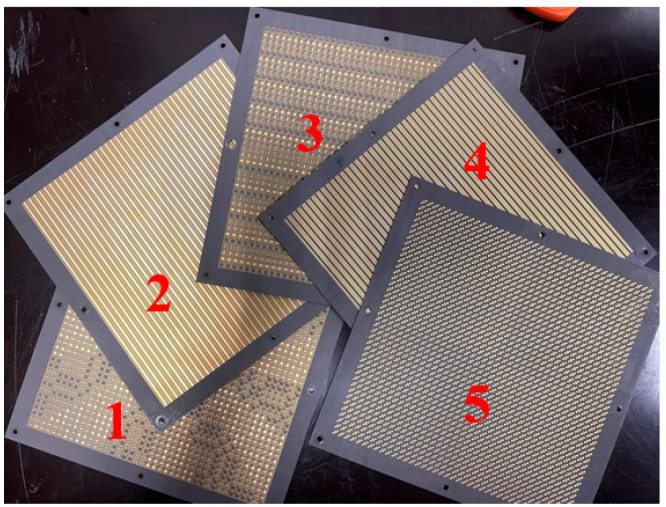
Five metasurfaces prototypes for cascading.

As shown in Figure 16, five metasurface prototypes are fabricated using PCB manufacturing processes according to the designed phase encoding schemes. Each metasurface measures 170 mm × 170 mm with eight 3 mm-diameter through-holes at the edges for cascaded mounting on custom fixtures. Specifically, the 1st and 3rd layers feature double-sided copper structures with grating layers on the back and phase encoding layers on the front; the 2nd and 4th layers are single-sided copper structures corresponding to different grating designs; and the 5th layer is a double-sided copper structure with a grating layer on the back and a polarization conversion layer on the front.

**Figure 17:**
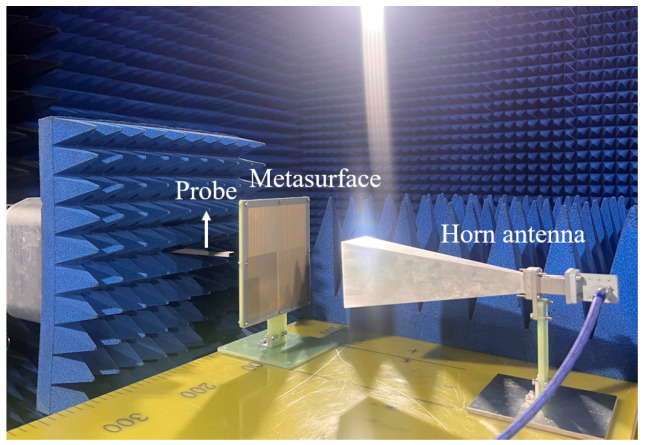
Near-field OAM test system in a microwave anechoic chamber.

To validate the effectiveness of the transmission OAM beam functions, a comprehensive simulation and experimental verification scheme is designed. The verification process is conducted in a microwave anechoic chamber by measuring near-field distributions in target regions to evaluate OAM beam characteristics. Considering the reciprocal nature of the *f*_1_ and *f*_4_ transmission functions, near-field distributions in both ±*z* half-spaces are measured to comprehensively demonstrate their performance. The measurement system configuration for transmission functions is shown in Figure 17, primarily consisting of a vector network analyzer (VNA), horn antenna, metasurface prototype, two-dimensional scanning platform, and supporting near-field testing control software. The metasurface prototype is mounted via custom fixtures. During testing, one VNA port connects to the horn antenna as the excitation source, while the other port connects to a probe as the receiver. The two-dimensional scanning platform controls the receiver probe’s movement trajectory across the detection plane, executing a systematic raster scan pattern to form a square scanning grid with predetermined spatial resolution. The probe moves in a step-by-step manner along both the x and y axes at uniform intervals, ensuring complete coverage of the measurement area and maintaining consistent sampling density throughout the scanning process. At each discrete measurement point, the VNA simultaneously captures both amplitude and phase information of the transmitted electromagnetic field through complex S-parameter measurements (S_21_). All measurement data undergoes real-time digital processing within the VNA, with both magnitude and phase components systematically stored and analyzed by the workstation control software for comprehensive beam characterization. The horn antenna serves as the excitation source, positioned 130 mm from the metasurface to excite first-order OAM transmission beams. The receiving probe is located 100 mm behind the metasurface, scanning a 150 mm × 150 mm near-field detection plane to obtain amplitude and phase distributions. Measurements in both ±*z* spaces are achieved by flipping the fixture, while *x* and *y* polarization data are obtained by rotating the probe.

**Figure 18:**
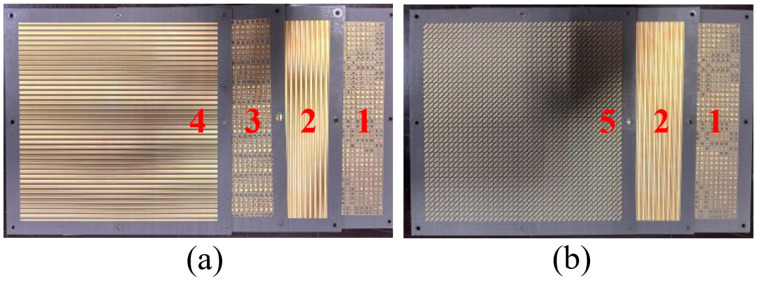
(**a**) Cascaded Module-A and Module-B (**b**) Cascaded Module-A and Module-C.

As shown in Figure 18, to realize the OAM beam generation functions *f*_1_ and *f*_4_, the fabricated metasurfaces need to be assembled in different modular configurations. Figure 18a shows the cascaded assembly scheme of Module-A and Module-B, where the 1st and 2nd metasurfaces are assembled to form the Module-A metasurface, and the 3rd and 4th metasurfaces are assembled to obtain Module-B. The two modules are secured together through 8 through-holes at the metasurface edges using 3 mm diameter screws. Figure 18b presents the cascaded assembly method of Module-A and Module-C by securing the 1st, 2nd, and 5th metasurface layers together.

**Figure 19:**
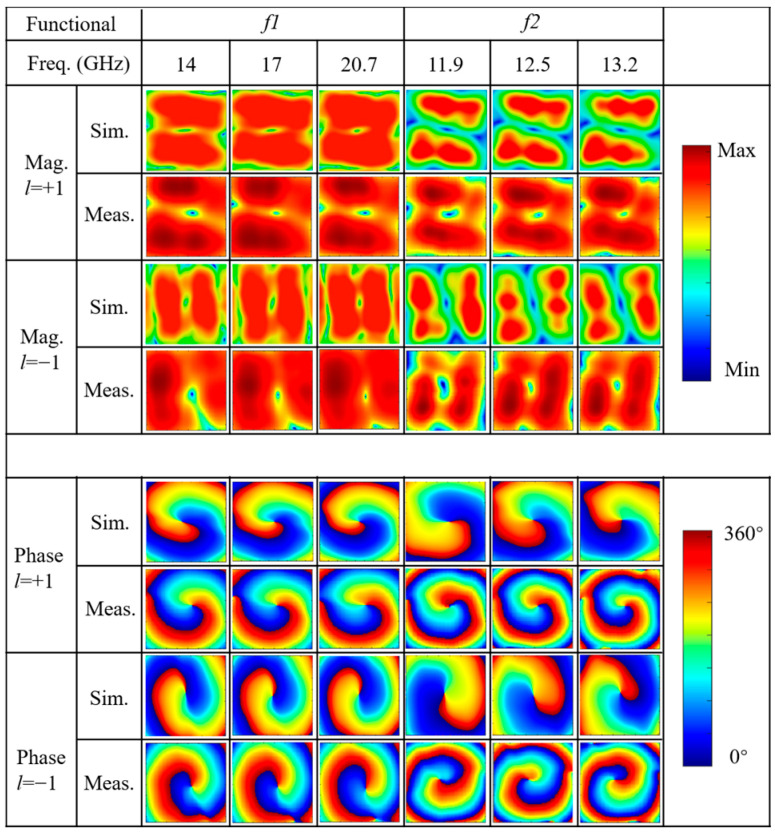
Comparison of simulated and measured electric field amplitude and phase distributions of OAM beams under two functional configurations.

To comprehensively evaluate the broadband performance of the two configurations, three representative frequency points within each operating bandwidth are selected for detailed analysis, corresponding to the lowest, center, and highest frequencies within each band. Figure 19 presents the comparison between simulated and measured electric field magnitude and phase distributions for functions *f*_1_ and *f*_4_ at all measurement frequency points. The testing encompasses full-space manipulation capability in ±*z* directions, where the −*z* space generates *l* = +1 OAM beams, and the +*z* space generates *l* = −1 OAM beams, achieving reciprocal co-polarized (*f*_1_) and cross-polarized (*f*_4_) transmission modes. From the magnitude distribution results, it can be observed that at all measurement frequency points, the electric field intensity exhibits the characteristic annular “doughnut” pattern, with near-zero energy in the central region and bright ring-shaped intensity distribution in the periphery, which is the typical signature of OAM beams. The radius and intensity of the annular structure remain relatively stable across different frequencies, indicating good consistency of OAM beam generation across the broad frequency bands. Regarding the phase distribution analysis, the results demonstrate excellent agreement between simulation and measurement across all frequency points. For *l* = +1 OAM modes, the phase patterns exhibit smooth counterclockwise helical wavefronts with azimuthal phase variation from 0° to 360° around the beam axis, creating the characteristic spiral phase structure with a single-arm spiral pattern. Conversely, for *l* = −1 OAM modes, the phase distributions show clockwise helical wavefronts with opposite azimuthal phase progression, forming mirror-symmetric spiral structures. The measured phase maps closely match the simulated predictions, with phase singularities clearly observable at the beam center where the phase becomes undefined due to zero field amplitude. The phase gradient consistency across different frequencies validates the broadband OAM generation capability.

**Figure 20:**
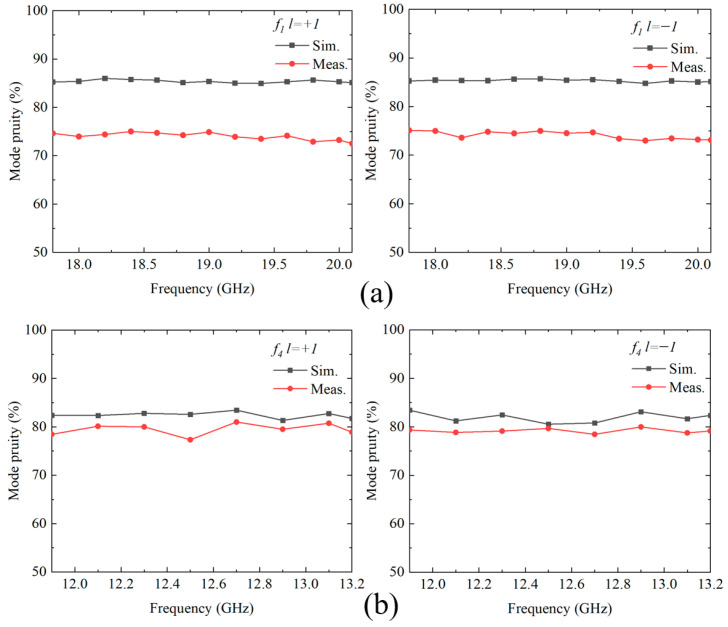
Mode purity analysis of OAM beams generated by two functional configurations. (**a**) Function *f*_1_ (14–20.7 GHz). (**b**) Function *f*_4_ (11.9–13.2 GHz).

To more accurately evaluate the quality of OAM beams generated by the metasurface within the operating frequency band, the mode purity at each frequency point is calculated. Mode purity is defined as the ratio of the dominant mode power to the total power of all the modes it possesses and is expressed in the following equation:

(2) $$Mode\;Purity=\frac{A_{l}^{2}}{\sum A_{i}^{2}}$$

where *A_i_* denotes the magnitude of the *i*-th mode. The ratio between the target OAM mode power and the total power of modes ranging from −9th to +9th order is computed. Figure 20 shows the spectral characteristics of the *l* = ±1 mode in the ±*z* space for both simulation and measurement results. The mode purity analysis demonstrates excellent performance across all functional configurations. For function *f*_1_, the simulation results achieve average mode purities of 85.89% and 85.87% for the *l* = +1 and *l* = −1 modes, respectively, while experimental results yield 78.67% and 79.62%, yielding a simulation-to-experiment deviation of only 6.25%. For function *f*_4_, the *l* = +1 OAM beam achieves simulated mode purity ranging from 81.32 to 83.45% (average 82.42%) with experimental results of 77.34–81.00% (average 79.38%), showing a deviation of approximately 3.04%. The *l* = −1 OAM exhibits simulated purity of 80.54–83.43% (average 81.95%) and experimental results of 78.45–79.98% (average 79.16%), with a deviation of approximately 2.79%. The good consistency between simulation and experimental results validates the reliability and practicality of the modular metasurface design approach.

**Figure 21:**
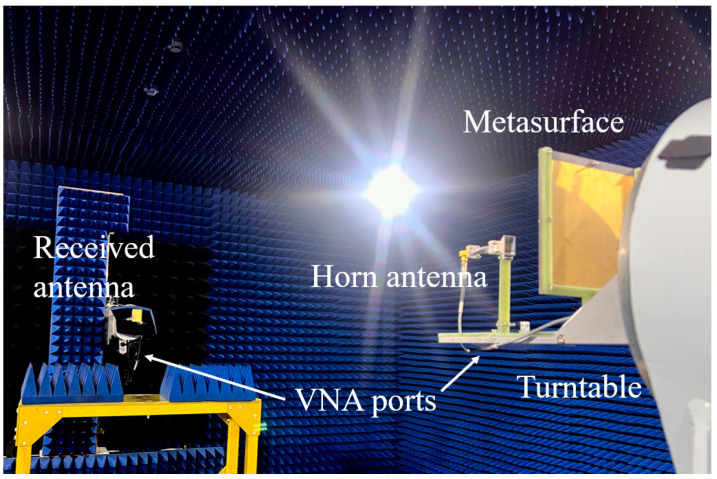
Far-field pattern testing system in a microwave anechoic chamber.

Having completed the simulation verification of the two transmission functions, the following section presents the measurement analysis of the three reflection functions. A far-field measurement system is established in the anechoic chamber, as illustrated in Figure 21. The far-field measurement system employs two horn antennas serving as the transmitting and receiving antennas, respectively. Both horn antennas are vertically positioned to enable co-polarized wave transmission and reception. The transmitting antenna is mounted together with the metasurface on a precision rotary stage, with the antenna positioned for normal incidence at the center of the metasurface at a separation distance of 150 mm. During measurements, the rotary stage rotates within an azimuth angle range of −90° to +90°, while the receiving antenna remains fixed at a distance of 3 m from the metasurface to measure the gain distribution at different angles.

**Figure 22:**
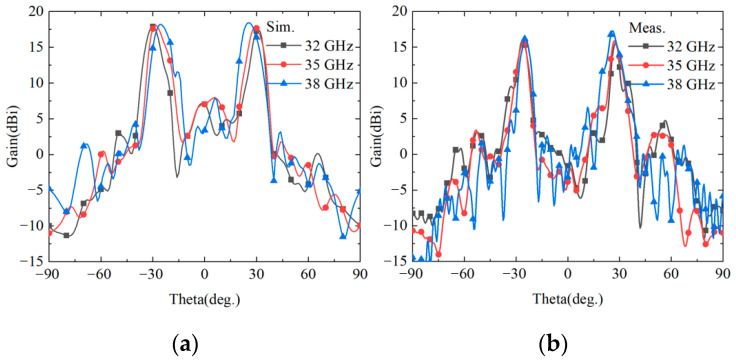
−*z* space reflection dual-beam simulation measurement results. (**a**) simulation results. (**b**) measurement results.

The simulation and experimental results for the reflected dual-beam function are presented in Figure 22. To thoroughly validate the stability of this function across the entire operating frequency band, two edge frequencies at 32 GHz and 38 GHz, along with the center frequency at 35 GHz within the frequency band, are selected as representative frequency points for analysis. The results demonstrate excellent consistency between simulation and experimental data. The two symmetric beams appear precisely at azimuth angles of ±30°, fully conforming to the design expectations. The simulation results show that the peak gains of the two symmetric beams vary within the range of 17.41–18.47 dBi, while the corresponding experimental results exhibit peak gains ranging from 15.58 to 16.79 dBi, with a deviation of approximately 1.7 dBi from the simulation results. As frequency increases, the gain shows an increasing trend while the beam steering angles slightly decrease, which is a normal phenomenon attributed to the inherent dispersive characteristics of the metasurface structure. The experimental results fully validate that the cascaded structure can generate reflected dual beams in the −*z* half-space within the Ka-band, confirming the effectiveness and practicality of the design approach.

**Figure 23:**
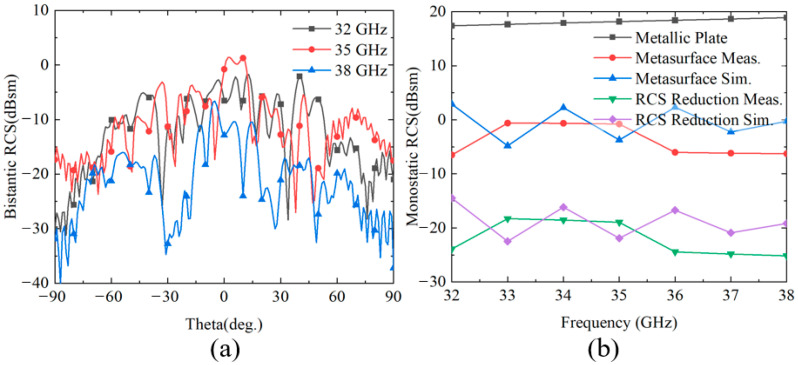
(**a**) Bistatic RCS test results at 32, 35, and 38 GHz. (**b**) Comparison of Monostatic RCS at each frequency point within the band and RCS reduction with a metal plane.

Figure 23 presents the simulation and experimental results of RCS and RCS reduction for function f_5_ in the +*z* space. To comprehensively evaluate the stealth performance of the metasurface, three representative frequency points at 32 GHz, 35 GHz, and 38 GHz are selected for bistatic RCS characteristic analysis, as shown in Figure 23a. The bistatic RCS measurement results demonstrate that the metasurface achieves significant low-RCS performance at all tested frequency points. Through phase manipulation, the scattered energy of incident waves is redistributed, dispersing the originally concentrated reflected energy to other directions, thereby effectively reducing the backscattering intensity. Figure 23b shows the monostatic RCS results from both simulation and measurement, along with the RCS reduction comparison against a metallic plate of identical dimensions. Within the 32–38 GHz frequency band, the measured monostatic RCS of the metasurface varies from −6.5 dBsm to −0.62 dBsm, while the RCS of the equivalent metallic plate ranges from approximately 17.4 to 18.89 dBsm, yielding a difference exceeding 18 dB. The metasurface achieves substantial RCS reduction compared to the metallic plate, with experimental results showing RCS reduction values ranging from −18.29 dBsm to −25.17 dBsm and an average reduction of approximately −22.04 dBsm. The simulation results demonstrate RCS reduction values from −14.5 dBsm to −22.49 dBsm, with an average reduction of approximately −18.84 dBsm. The simulation and experimental results maintain good consistency in terms of variation trends and frequency response characteristics, validating the correctness of the theoretical design. However, there exists approximately a 3–4 dB discrepancy in specific numerical values, which is primarily attributed to the combined effects of geometric dimensional deviations during actual fabrication, dispersive characteristics of material parameters, and multipath effects in the measurement environment. Despite these differences, the comparison between experimental and simulation results sufficiently demonstrates the excellent stealth performance of the metasurface structure in the +*z* space.

**Figure 24:**
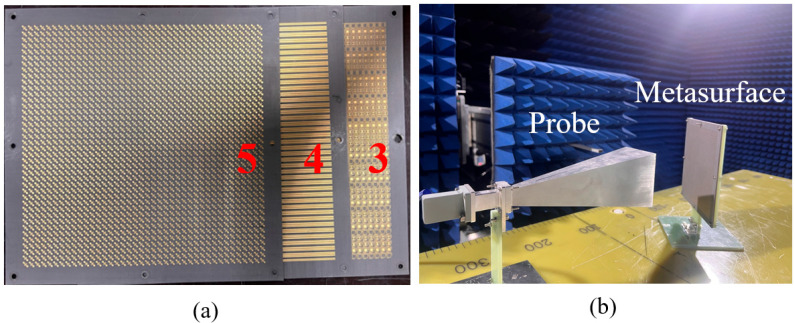
(**a**) Schematic diagram of assembling cascaded Module-B and Module-C. (**b**) Near-field measurement system and setup.

For the linear-to-circular polarization conversion function of *f*_5_ in the −*z* space, Module-B needs to be cascaded with Module-C by assembling the fabricated metasurfaces 3, 4, and 5, as shown in Figure 24a. Near-field scanning of amplitude and phase is employed to verify the correctness of this function. The measurement setup and system configuration are illustrated in Figure 24b, where the feed horn antenna is vertically positioned to illuminate the metasurface at oblique incidence. The probe is positioned in both horizontal and vertical orientations to collect the near-field amplitude and phase for vertical and horizontal polarizations, respectively. The probe is positioned 200 mm from the metasurface, the feed horn center is located 130 mm from the metasurface center, and the probe scanning area covers 100 mm × 100 mm.

**Figure 25:**
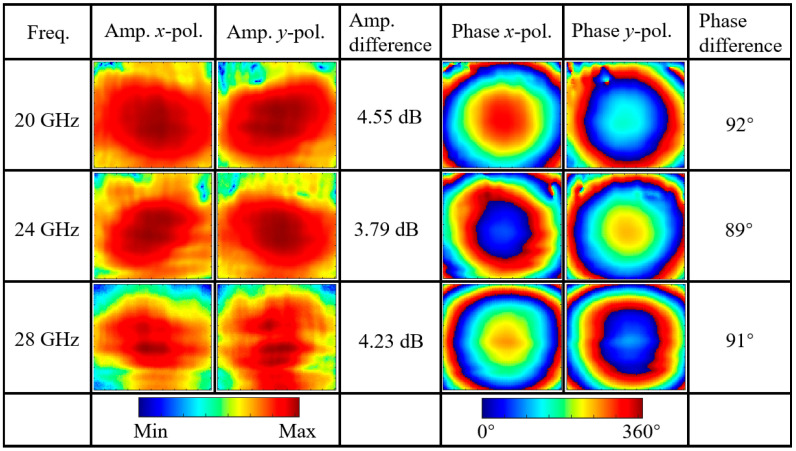
Near-field measurement results of linear-to-circular polarization conversion at representative frequencies (20 GHz, 24 GHz, and 28 GHz): amplitude and phase distributions of orthogonal polarization components.

Figure 25 presents the measurement results, with three representative frequencies at 20 GHz, 24 GHz, and 28 GHz selected to demonstrate the functional performance of the metasurface across the entire operating frequency band. From the amplitude distribution obtained through near-field scanning, it can be observed that at all tested frequency points, both *x*-polarized and *y*-polarized components exhibit excellent symmetric distribution in the central region, with energy predominantly concentrated at the center of the scanning area. This indicates that the metasurface can effectively convert the incident linearly polarized wave into reflected beams with well-defined spatial distribution.

Through detailed analysis of the central region in the scanned field distribution, the key characteristics of linear-to-circular polarization conversion can be clearly observed. In terms of amplitude, the amplitude differences between *x*-polarized and *y*-polarized components in the central region are 2.55 dB, 1.79 dB, and 2.23 dB at the three frequency points, respectively, indicating that the amplitude ratio of the two orthogonal polarization components approaches the ideal condition. More importantly, the phase distribution results show that the phase differences between the two orthogonal polarization components are 92°, 89°, and 91°, respectively, all very close to the 90° phase difference required for ideal circular polarization. These measurement data fully demonstrate that the metasurface successfully achieves circular polarization conversion functionality.

## 4. Conclusions

This paper presents a novel modular cascading approach for multifunctional metasurfaces based on three distinct multilayer modules. The cascading is accomplished through direct attachment assembly, avoiding air coupling effects that could compromise system performance. Through cascading configurations, the design balances coupling impedance conditions between modules and establishes electromagnetic coupling between resonators, thereby enabling broadband operation. By combining the three modules in pairwise configurations, this approach maximizes the utilization of each individual module, achieving the maximum number of operational channels. The proposed system realizes comprehensive electromagnetic wavefront manipulation across four broadband frequency ranges, demonstrating diverse functionalities including orbital angular momentum beam generation, polarization conversion, beam splitting, and radar cross-section reduction with seven operational channels spanning both transmission and reflection modes. Experimental validation confirms excellent agreement between simulated and measured results across all operational channels. Table 1 lists the research on multi-functional and multi-channel metasurfaces published in the last two years. As can be seen, previous studies have achieved excellent performance in specific functions or frequency bands, such as achieving ultra-wide bandwidth or multiple channel numbers. However, they cannot simultaneously satisfy multi-band, broadband, and full-space transmission/reflection control requirements. The modular cascaded metasurface proposed in this study effectively advances the development of such multi-objective demands, achieving multi-functionality, multi-channel, multi-frequency, and full-space transmission/reflection control. This modular architecture provides a robust and efficient solution for multifunctional electromagnetic wave control applications.

## Figures and Tables

**Table 1:** Performance comparison of the proposed metasurfaces with previous works.

Ref.	Channels	Frequency (GHz)	Bandwidth (%)	Transmission/Reflection
[34]	3	8.11/15/23	23.7/26.7/17.3	R
[35]	3	8/12	NA	T/R
[36]	4	8.7/15.8	NA	T/R
[37]	8	12.55/29	NA	R
[38]	3	20/38.75	40/6.5	T/R
This work	7	12.55/17.35/24/36	10.4%/38.6/33.3/16.7	T/R

## Data Availability

The original contributions presented in this study are included in the article. Further inquiries can be directed to the corresponding author.
